# The renoprotective effect of curcumin against cisplatin-induced acute kidney injury in mice: involvement of miR-181a/PTEN axis

**DOI:** 10.1080/0886022X.2020.1751658

**Published:** 2020-04-25

**Authors:** Si-Jia Huang, Jing Huang, Yun-Bo Yan, Jiao Qiu, Rui-Qiao Tan, Yu Liu, Qing Tian, Li Guan, Shuai-Shuai Niu, Yanxiang Zhang, Zhijiang Xi, Ying Xiang, Quan Gong

**Affiliations:** a Department of Cell Biology and Genetics, School of Basic Medicine, Health Science Center, Yangtze University, Jingzhou, China; b Laboratory of Oncology, Center for Molecular Medicine, School of Basic Medicine, Health Science Center, Yangtze University, Jingzhou, China; c Department of Immunology, School of Basic Medicine, Health Science Center, Yangtze University, Jingzhou, China; d Clinical Molecular Immunology Center, Health Science Center, Yangtze University, Jingzhou, China

**Keywords:** Curcumin, cisplatin, AKI, miR-181a, PTEN, renoprotective

## Abstract

**Background:**

Nephrotoxicity, especially acute kidney injury (AKI), is the main dose-limiting toxicity of cisplatin. Although recent studies showed that curcumin prevented cisplatin-induced AKI effectively, further studies to understand the mechanism are required.

**Methods:**

We established an AKI mouse model. Male C57BL/6 mice were assigned to three groups: saline group (control), cisplatin group (CP), and curcumin + cisplatin group (CP + Cur). The CP group received a single intraperitoneal (i.p.) injection of cisplatin, while the control group received saline. The CP + Cur group received i.p. curcumin three days before cisplatin injection and curcumin administered for another three days until the day before euthanization. Renal injury was assessed by serological and histological analysis. Western blotting and quantitative real-time polymerase chain reaction (qRT-PCR) were used to detect the phosphatase and tensin homolog (PTEN), and microRNA (miR)-181a expression in the renal tissues. Bioinformatics prediction and western blotting methods validated the targets of miR-181a *in vitro*.

**Results:**

Curcumin treatment alleviated cisplatin-induced nephrotoxicity as validated by the blood urea nitrogen (BUN) values, and histological analysis of kidneys. At the molecular level, curcumin treatment decreased miR-181a expression level, which was induced by cisplatin and restored the *in vivo* expression of PTEN, which was suppressed by cisplatin. We verified the direct regulation of PTEN by miR-181a in cultured human embryonic kidney 293T cells.

**Conclusions:**

We showed the involvement of miR-181a/PTEN axis in the renoprotective effect of curcumin against cisplatin-induced AKI, and provide new evidence on the ability of curcumin to alleviate cisplatin-induced nephrotoxicity.

## Background

Cisplatin has a wide range of anti-tumor effects and has been used to treat various malignant tumors [1]. However, nephrotoxicity is the main dose-limiting toxicity, which is characterized by renal tubular cell necrosis, tissue damage, renal dysfunction, and acute kidney failure [2]. Although various measures have been taken to prevent renal injury, nearly 4–23% of patients are affected by acute kidney injury (AKI) [3]. The known mechanisms of cisplatin-induced renal injury include apoptosis, autophagy, oxidative stress, and inflammatory response due to direct injury to renal tubular epithelial cells and renal vessels [4].

Curcumin is the most important pharmacologically active component extracted from Curcuma. Curcumin has anti-inflammatory, anti-oxidative, oxygen-free radical scavenging, anti-fibrotic, and anti-cancer activities [5]. Recent studies reported that curcumin could prevent cisplatin-induced AKI effectively [6]. Ortega-Dominguez et al. showed that curcumin could protect cisplatin-induced AKI by preventing changes in mitochondrial bioenergy, ultrastructure, redox balance dynamics, and sirtuin-3 (SIRT3) expression levels [7]. Another study showed that curcumin reduced cisplatin-induced renal toxicity by suppressing renal tubular cell apoptosis [8]. In addition, a recent study showed that curcumin reduced cisplatin-induced nephrotoxicity by decreasing the expression of pro-inflammatory cytokines, including tumor necrosis factor (TNF)-α, interleukin (IL)-6 and IL-8 [9]. However, curcumin exerts a complex pharmacological effect, and its underlying renoprotective mechanism needs further investigation.

MicroRNAs (miRs) are non-coding RNAs of approximately 22 nucleotides in length, which induce mRNA degradation or inhibit protein synthesis by binding to specific sites of target mRNAs, thus playing a role in many physiological and pathological processes [10]. Some miRs are involved in the protective effect of curcumin against renal injury [11]. The phosphatidylinositol-3-kinase (PI3K)/AKT pathway is an important intracellular signal transduction pathway, regulating cell growth, cell cycle, apoptosis, migration, and other processes. PTEN can negatively regulate AKT activity [12]. It was shown that decreased expression of PTEN protein could reduce apoptosis of renal tubular epithelial cells [13].

In this study, we investigate the role of miRs and PTEN in renoprotective effect of curcumin against cisplatin-induced AKI in mice. It is aimed to reveal the mechanism of curcumin which protected the kidney and provided evidence on the therapeutic application of curcumin in alleviating AKI.

## Materials and methods

### Drugs

Curcumin (>97% purity) and cisplatin (>98.5% purity) used in this study were purchased from Meilunbio (Dalian, China). Both curcumin and cisplatin were dissolved in saline at 1 mg/mL before use.

### Mouse model of AKI and treatments

Male C57BL/6 mice (8–10 weeks old and weighing 20–25 g) were purchased from the Experimental Animal Center of the Three Gorges University (no. 2017-0012, Yichang, China). The animals were caged in a room with suitable temperature and illumination, and free to access food and water. The mice were grouped with eight mice per group as follows: saline control (control), cisplatin injury group (CP), and curcumin treatment group (CP + Cur). The CP group received a single intraperitoneal (i.p.) injection of cisplatin (20 mg/kg/day per mouse), while the control group was injected with an equal volume of saline. The CP + Cur group received i.p. curcumin (20 mg/kg/day per mouse) for three days before injection with cisplatin (20 mg/kg/day per mouse), and curcumin administered for another three days until one day before euthanization. All animals were euthanized three days after cisplatin treatment. The protocol for cisplatin-induced AKI [14] and curcumin treatment [15] was based on previous publications. All animal experiments were following the protocols approved by the International Animal Care and Use Committee (IACUC) at Yangtze University.

### Serological experiments

The mouse blood was extracted, and serum was separated from blood by centrifugation. Blood urea nitrogen (BUN) was measured immediately by urea assay kit from Jiancheng Bioengineering Institute (Nanjing, China) following the instructions.

### Histological analysis

After the mice were euthanized, their kidneys were carefully separated. One kidney from each mouse was subjected to histological analysis, and the other was extracted to isolate ribonucleic acid (RNA) and proteins. Kidneys were washed with phosphate-buffered saline (PBS) and fixed in 4% paraformaldehyde. After dehydration and embedding, they were sliced. The sections were stained with hematoxylin–eosin (HE) solution. The general morphology of the kidney tissues was visualized under the microscope. The renal histological damage was scored based on the percentage of damaged tubules as follows: 0, no damage; 1, less than 25%; 2, 25–50%; 3, 50–75%; and 4, more than 75% [14].

### Quantitative real-time polymerase chain reaction (qRT-PCR)

Total RNA was extracted from the kidney using TRIzol (Beyotime, Beijing, China). The RNA was reverse transcribed to cDNA by Transcriptor First Strand cDNA Synthesis Kit (Roche, Mannheim, Germany) following the manufacture’s protocol. A stem-loop specific primer was used for the synthesis of miR-181a cDNA. qRT-PCR was performed with the SYBR Green master mix (Roche, Mannheim, Germany) using the Applied Biosystems PCR system (Applied Biosystems, Darmstadt, Germany). The sequences of primers (Sangon, Shanghai, China) used in qRT-PCR are listed in Table 1. The miR-181a expression levels were normalized to U6. The expression levels of PTEN were normalized to glyceraldehyde-3-phosphate dehydrogenase (GAPDH).

**Table 1.** The sequences of primers for real-time PCR.

Gene	Primer sequence (5′–3′ end)
PTEN-F	TTCCTGCAGAAAGACTTGAAGG
PTEN-R	AAGGATACTGTGCAACTCTGC
GAPDH-F	ACTCAGGAGAGTGTTTCCTCG
GAPDH-R	TTTGCCGTGAGTGGAGTCAT
miR-181-F	CCCAACATTCAACGCTGTC
miR-181-R	AGTGCGTGTCGTGGAGT
U6-F	GCTTCGGCAGCACATATACTAAAAT
U6-R	CGCTTCACGAATTTGCGTGTCAT

### Western blotting

The proteins in the renal tissues were extracted with lysis buffer. After denaturation, 40 μg of protein was loaded on the 10% sodium dodecyl sulfate (SDS)-polyacrylamide gel and then transferred to polyvinylidene fluoride membranes. After blocking overnight at 4 °C with 4% skimmed milk, the membranes were incubated with the anti-GAPDH (Multi Sciences, Hangzhou, China) and anti-PTEN (Multi Sciences, Hangzhou, China) antibodies. After washing, the membranes were incubated with the horseradish peroxidase-conjugated secondary antibody (Multi Sciences, Hangzhou, China) to identify the respective proteins. The bands were visualized using enhanced chemiluminescence (Meilun, Shanghai, China) and quantified with Image J software. GAPDH was used as an internal control.

### Cell culture and transfection

The human embryonic kidney 293T cell line was conserved in our laboratory. The cells were cultured in Dulbecco’s modified Eagle medium (Invitrogen, ‎Waltham, MA) with 10% fetal bovine serum and 1% penicillin/streptomycin in a humidified atmosphere containing 5% CO_2_ at 37 °C. Lipofectamine 2000 (Invitrogen, ‎Waltham, MA) was used for transfecting miR-181a mimic oligonucleotides (sequence 5′-AACAUUCAACGCUGUCGGUGAGU-3′) or the negative control (NC) (Gene Pharma Company, Shanghai, China), targeting none of the mRNAs.

### Statistical analysis

Data were presented as the means ± standard deviation. One-way analysis of variance (ANOVA) following a *post hoc* test was applied to reveal the differences between groups. *p <* .05 was considered significant.

## Results

### Curcumin alleviates cisplatin-induced mouse acute kidney injury

**Figure 1.**
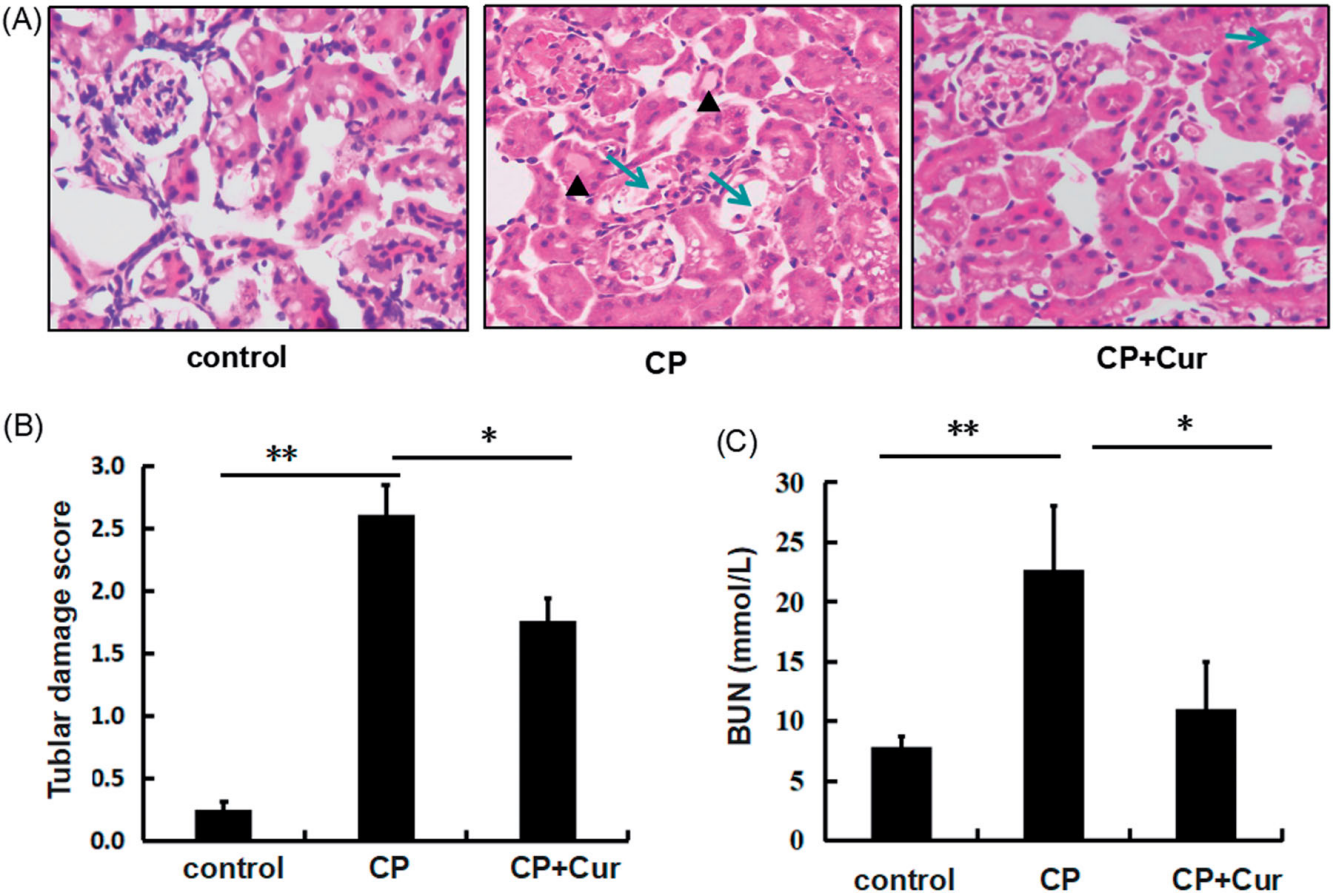
The evaluation of renal damage in mice. (A) Histopathological analysis of the renal cortex of mice by hematoxylin and eosin (HE) staining (×200). The triangles and arrows indicate intratubular cast formation and damaged tubular cells, respectively. (B) Semi-quantitative analysis of histological appearance, one-way ANOVA followed by the Bonferroni test; (C) the values of serum BUN in mice, analyzed by one-way ANOVA followed by Games-Howell test. The mice were treated with saline (control): *n* = 5, cisplatin (CP): *n* = 6, cisplatin and curcumin (CP + Cur): *n* = 6. **p < .01, *p < .05.

We investigated the effect of curcumin in a mouse model of cisplatin-induced AKI by assessing the renal damage and renal function by histopathological and serological analysis. Results showed, displayed tubular cavity expansion, intratubular cast formation, and tubular epithelial cell necrosis in the cortex of mouse kidneys from the CP group, while the control group showed normal glomerular and renal tubule structures, and no renal tubular epithelial cell necrosis (Figure 1(A,B)). BUN values increased in the CP group compared to control (*p <* .01) (Figure 1(C)).

Curcumin treatment reduced cisplatin-induced renal tubular damage significantly in the CP + Cur group compared to the CP group (Figure 1(A,B)). BUN values decreased substantially in CP + Cur group compared to the CP group (*p <* .05) (Figure 1(C)). The values of BUN and histological damage scores are listed in Table 2. These results suggested that curcumin played a renoprotective role in cisplatin-induced AKI, which was consistent with previous publications on the attenuation of renal injury by curcumin [8,16].

**Table 2.** Curcumin effects on blood urea nitrogen (BUN) and renal tubular damage.

Groups	BUN (mmol/L)	Tubular damage sore
Control (*n* = 6)	7.79 ± 0.88	0.25 ± 0.06
CP (*n* = 5)	22.63 ± 5.38^a^^,^**	2.61 ± 0.24^a^^,^*
CP + Cur (*n* = 5)	11.05 ± 3.87^b^^,^*	1.76 ± 0.18^b^^,^*

BUN: one-way ANOVA followed by Games-Howell test. Tubular damage sore: one-way ANOVA followed by the Bonferroni test.

* *p* < .05.

** *p* < .01.

^a^ Statistically significant compared to control.

^b^ Statistically significant compared to cisplatin group.

### Curcumin decreases miR-181a expression in cisplatin-treated mice kidneys

**Figure 2.**
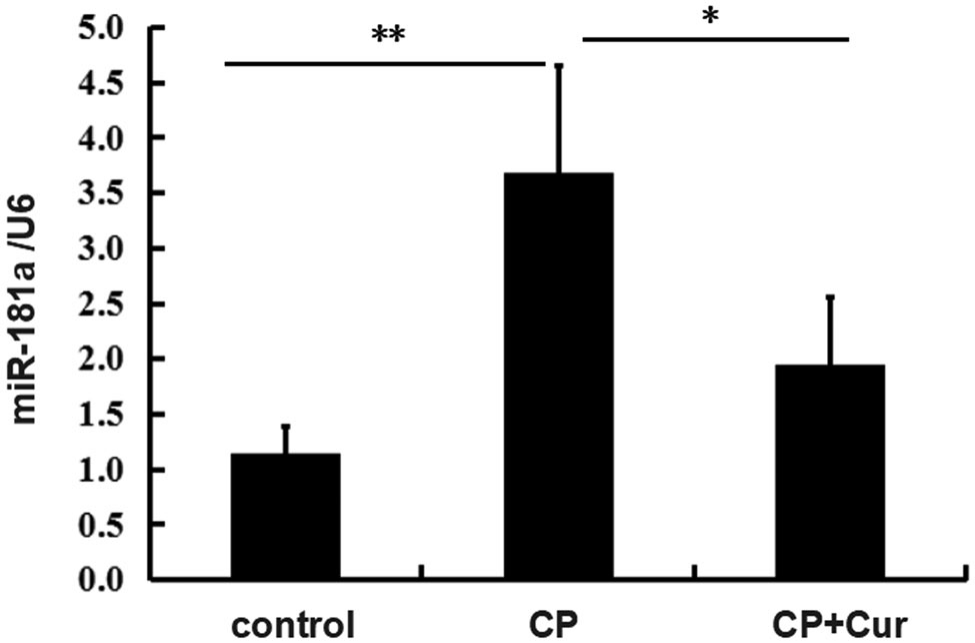
The miR-181 expression in the kidneys of mice treated with saline (control), cisplatin (CP), cisplatin, and curcumin (CP + Cur). The levels of miR-181 were measured by quantitative real-time PCR (qRT-PCR). U6 was used as the reference gene. Statistical significance was analyzed by one-way ANOVA followed by the Bonferroni test. **p < .01, *p < .05.

Some miRs were reported to be involved in the renoprotective effect of curcumin against renal injury [11]. miR-181a, a member of miR-181 families, was reported to increase renal blood flow during acupuncture with low-frequency electrical stimulation [17]. Our results showed markedly increased miR-181a in the CP group compared to the control group as assessed by qRT-PCR. miR-181a was decreased in CP + Cur group compared to the CP group (Figure 2). The miR-181a expression data after curcumin treatment are listed in Table 3. The data showed that curcumin inhibited miR-181a expression.

**Table 3.** Curcumin effects on gene expression.

Groups	MiR-181a/U6	PTEN protein/GAPDH	PTEN mRNA/GAPDH
Control (*n* = 6)	1.14 ± 0.25	1.05 ± 0.10	1.27 ± 0.31
CP (*n* = 5)	3.69 ± 0.96^a^^,^**	0.60 ± 0.20^a^^,^*	0.942 ± 0.92
CP + Cur (*n* = 5)	1.95 ± 0.61^b^^,^*	0.99 ± 0.10^b^^,^*	8.27 ± 5.74^b^^,^*

One-way ANOVA followed by the Bonferroni test.

* *p* < .05.

** *p* < .01.

^a^ Statistically significant compared to control.

^b^ Statistically significant compared to cisplatin group.

### Curcumin restores PTEN expression in cisplatin-treated mice kidneys

It was shown that decreased expression of PTEN protein could reduce apoptosis of renal tubular epithelial cells [13]. Moreover, Zhang et al., using immunostaining and western blotting assay, showed increased PTEN protein level in the renal cortical tubular cells of mice treated with cisplatin [18].

**Figure 3.**
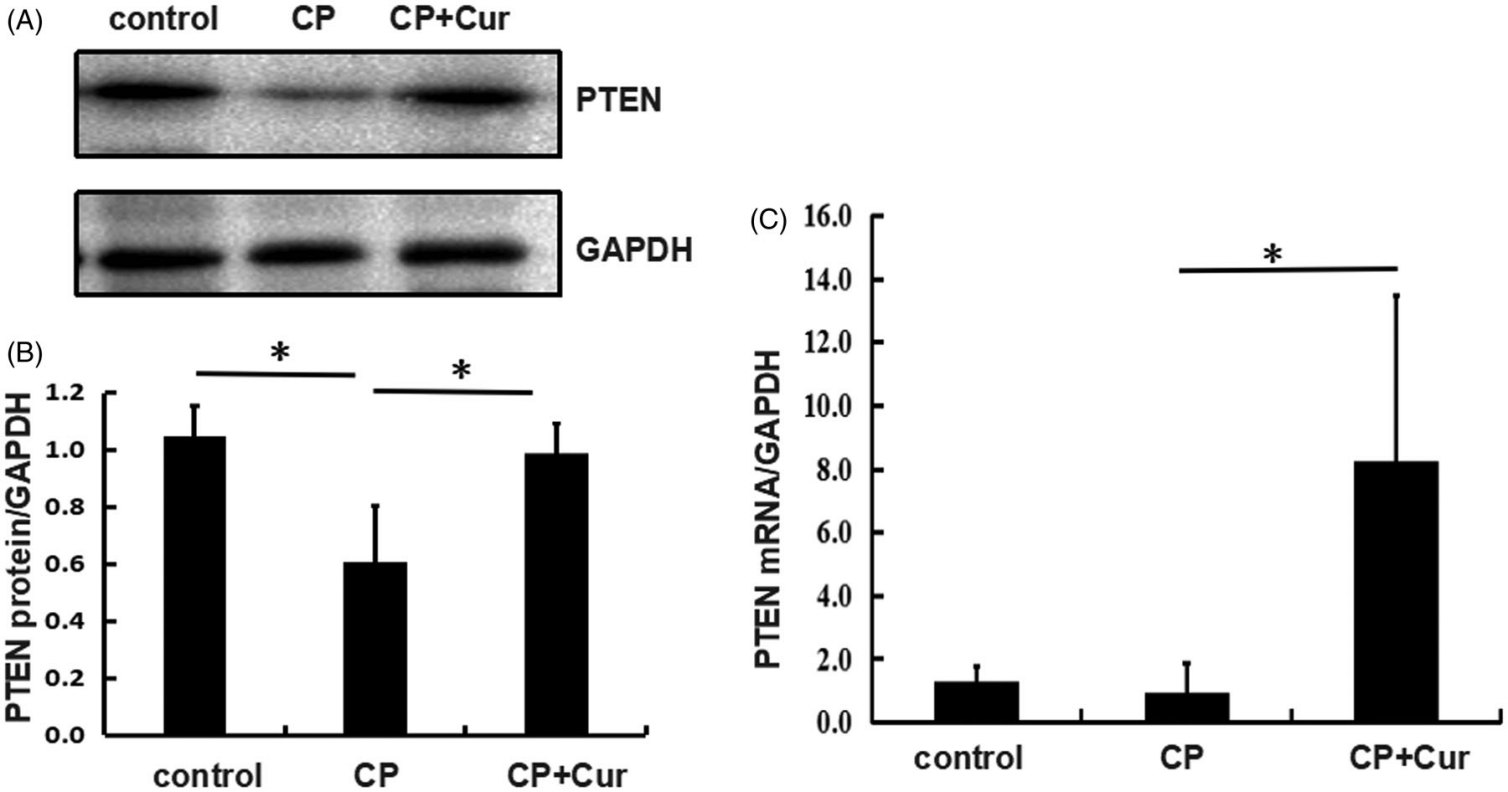
PTEN expression in the kidneys of mice treated with saline (control), cisplatin (CP), cisplatin, and curcumin (CP + Cur). (A) PTEN protein levels measured by western blotting. GAPDH was used as the reference gene. (B) The quantitative analysis of protein levels (normalized to GAPDH) by Image J. (C) PTEN mRNA levels measured by quantitative real-time PCR (qRT-PCR). GAPDH was used as the reference gene. Statistical significance was analyzed by one-way ANOVA followed by the Bonferroni test. *p < .05.

To investigate how curcumin regulates PTEN expression, we examined PTEN levels in kidneys of mice treated with cisplatin alone or in combination with curcumin. Our results showed a downregulated PTEN protein level in the CP group compared to the control group (Figure 3(A,B)). However, PTEN mRNA level did not show a statistically significant decrease in the CP group compared to the control group (Figure 3(C)) and could be due to the high standard deviation in the CP group. Curcumin treatment restored the expression of PTEN both at protein and mRNA levels, in CP + Cur group in comparison to the CP group (Figure 3), and showed an opposite trend in miR-181a expression (Figure 2). The data for curcumin effects on gene expression are presented in Table 3. These results showed that curcumin restored PTEN expression in mice treated with cisplatin.

### Direct regulation of PTEN by miR-181a *in vitro*

**Figure 4.**
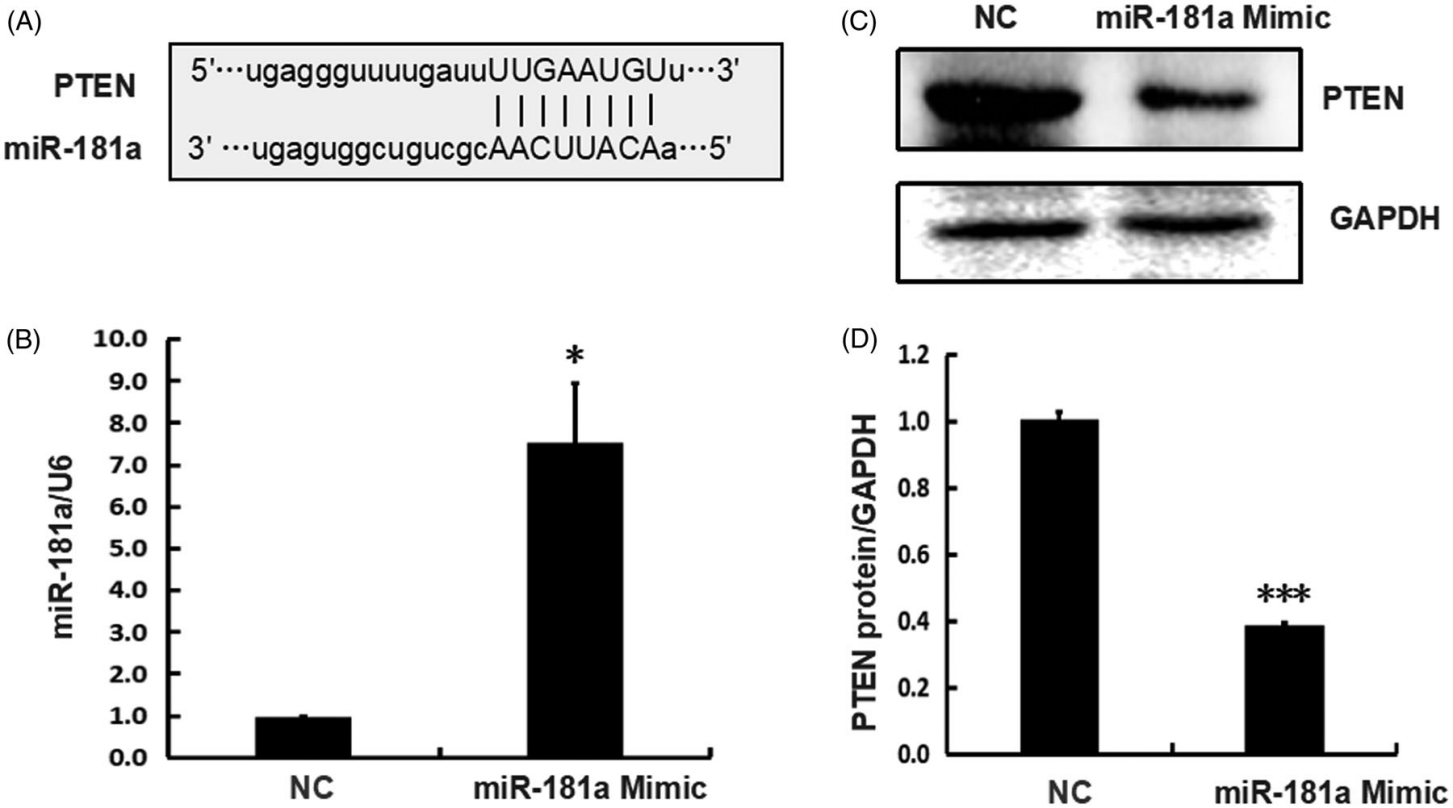
PTEN is the direct target of miR-181 *in vitro.* (A) The base-pairing sites of miR-181 and the 3′ UTR of PTEN mRNA, as predicted by bioinformatics software. (B) The miR-181 expression measured by quantitative real-time PCR (normalized to U6) 48 h after transfection with miR-181 mimic or NC in 293T cells. (C) The PTEN protein levels measured by western blotting 48 h after transfection with miR-181 mimic or NC in 293T cells. GAPDH was used as the reference gene. (D) A quantitative analysis of protein levels normalized to GAPDH was by Image J software. Statistical significance analysis was by the two-tailed Student’s *t*-test.

PTEN is a potential target of miR-181a as predicted by the bioinformatics database (PITA, PicTar, TargetScan) (Figure 4(A)). Jiang et al. [19] and Wu et al. [20] verified miR-181a ability to directly target the 3′UTR of PTEN in osteosarcoma cell line SAOS2 cells and primary chondrocytes obtained from osteoarthritis patients, respectively. However, the targets regulated by miRs may vary with different cell types. We investigated whether miR-181a regulates PTEN in the kidney tissue by transfecting the miR-181a mimic into cultured human embryonic kidney 293T cells and harvesting the cells 48 h later. The total RNA was extracted to analyze miR-181a expression levels. The proteins were extracted for analysis of PTEN protein levels. We showed the effect of the synthetic miR-181a mimic on increasing miR-181a expression levels (Figure 4(B)), in comparison to the NC treated cells. The PTEN protein level was downregulated significantly after transfection with miR-181a mimic compared to NC (Figure 4(C,D)). The data for PTEN regulation by miR-181a are listed in Table 4. The data showed the regulation of *in vitro* PTEN expression by miR-181a.

**Table 4.** Effect of miR-181a transfection of 293T cells on PTEN expression.

Transfection groups	MiR-181a/U6	PTEN protein/GAPDH
NC (*n* = 3)	0.945 ± 0.06	1.01 ± 0.02
miR-181a mimic (*n* = 3)	7.52 ± 1.43*	0.39 ± 0.01***

Two-tailed Student’s *t*-test.

* *p* <. 05, statistically significant compared to NC.

*** *p* <. 001, statistically significant compared to NC.

## Discussion and conclusions

Curcumin exerts anti-inflammatory, anti-oxidative, oxygen-free radical scavenging, and anti-fibrotic effects [5]. In renal diseases, curcumin prevented renal fibrosis by reducing the recruitment of M1 inflammatory macrophages by blocking the monocyte chemoattractant protein-1/CC chemokine receptor 2 (MCP-1/CCR2) pathway [21], inhibited the activation of nuclear factor kappa-B (NF-κB) in streptozotocin-induced diabetic nephropathy rats, and reduced pro-inflammatory cytokines: TNF-α and IL-1β [22]. In rats with glomerulonephritis, curcumin showed dose-dependent anti-fibrotic effects through the induction of home oxygenase 1 [23]. However, the mechanism underlying the attenuation of cisplatin-induced nephrotoxicity by curcumin is not understood fully.

Recent studies showed the involvement of miRs in various renal pathologies such as renal interstitial fibrosis, diabetic nephropathy, and ischemia/reperfusion injury (IRI) [24–26]. Suppression of miR-29 expression was shown to be responsible for the induction of collagen expression and renal interstitial fibrosis via transforming growth factor-β1 (TGF-β1) [24]. Increased miR-192 contributed to the glomerular injury in diabetes [25]. In addition, miR-21 could alleviate renal injury caused by ischemia-reperfusion [26]. Several studies showed that curcumin could alter miRs expression profiles in various tissues or cells [27,28]. Some miRs were involved in mediating the protective effect of curcumin against renal injury. Curcumin exerted a renoprotective effect against IRI by enhancing miR-146a expression [11]. In this study, we showed induction of miR-181a by cisplatin, and curcumin treatment decreased miR-181a levels, thus suggesting that miR-181a might be responsible for the renoprotective effect of curcumin.

PTEN is a negative regulator of AKT signaling pathway [12]. Recent studies indicated that PTEN played a role in kidney disease. Loss of PTEN expression contributes to increased TGF-β signaling and renal fibrosis [29]. In addition, deletion of PTEN in mouse proximal tubule, caused hypertrophy of renal proximal tubule cells [30]. In this study, PTEN expression was downregulated in renal tissues after cisplatin treatment and restored by curcumin treatment. These data indicated that PTEN played a role in the renoprotective effects of curcumin. PTEN was a validated target of miR-181a in osteosarcoma cell line SAOS2 cells and primary chondrocytes. We observed that *in vitro* expression of miR-181a inhibited PTEN protein expression in cultured human embryonic kidney 293T cells. However, *in vivo*, an opposite trend was observed between PTEN expression and miR-181a in mice (Figures 2 and 3). In this study, we could only verify one miR-181a target in mice and cultured cells due to the unavailability of samples from patients with AKI. We analyzed the data from the ENCORI (The Encyclopedia of RNA Interactomes) database to validate that PTEN was a target of miR-181a in human renal tissues. We observed a negative correlation between PTEN and miR-181a in the renal cancer samples (Table 5).

**Table 5.** The negative correlation between the expression of PTEN and miR-181a in renal cancer samples.

Cancer types	Number of samples	Coefficient *R*	*p* Value
Kidney renal clear cell carcinoma	517	–0.271	<.001
Kidney renal papillary cell carcinoma	289	–0.386	<.001

The data were obtained from database ENCORI (The Encyclopedia of RNA Interactomes).

In addition to PTEN, more miR-181a targets might mediate the effect of curcumin. It was reported that miR-181a promoted endogenous apoptotic pathways by targeting B cell lymphoma/leukemia-2 in cytosine arabinoside-resistant leukemia cells [31], and miR-181b increased the sensitivity of leukemia cells to chemotherapeutic drugs via targeting high mobility group protein and myeloid cell leukemia 1 [32]. We believe that the miR-181a/PTEN axis might play a significant role in the renoprotective effect of curcumin against cisplatin-induced AKI.

In conclusion, we revealed that curcumin exerted a renoprotective effect by downregulating miR-181a and upregulating PTEN expression. This study provides evidence on the therapeutic application of curcumin in alleviating AKI.

## Abbreviations

AKI: acute kidney injury
BUN: blood urea nitrogen
PTEN: phosphatase and tensin homolog
miR: microRNA
cDNA: complementary DNA
RNA: ribonucleic acid
qRT-PCR: quantitative real-time polymerase chain reaction
GAPDH: glyceraldehyde-3-phosphate dehydrogenase
IL: interleukin
TGF-β: transforming growth factor-β
